# Agreement between self-reported and physically verified male circumcision status in Nyanza region, Kenya: Evidence from the TASCO study

**DOI:** 10.1371/journal.pone.0192823

**Published:** 2018-02-12

**Authors:** Elijah Odoyo-June, Kawango Agot, Edward Mboya, Jonathan Grund, Paul Musingila, Donath Emusu, Leonard Soo, Boaz Otieno-Nyunya

**Affiliations:** 1 Division of Global HIV & TB (DGHT), U.S. Centers for Disease Control and Prevention (CDC), Kisumu, Kenya; 2 Impact Research and Development Organization, Kisumu, Kenya; 3 Division of Global HIV & TB (DGHT), U.S. Centers for Disease Control and Prevention (CDC), Atlanta, Georgia, United States of America; Cardiff University, UNITED KINGDOM

## Abstract

**Background:**

Self-reported male circumcision (MC) status is widely used to estimate community prevalence of circumcision, although its accuracy varies in different settings depending on the extent of misreporting. Despite this challenge, self-reported MC status remains essential because it is the most feasible method of collecting MC status data in community surveys. Therefore, its accuracy is an important determinant of the reliability of MC prevalence estimates based on such surveys. We measured the concurrence between self-reported and physically verified MC status among men aged 25–39 years during a baseline household survey for a study to test strategies for enhancing MC uptake by older men in Nyanza region of Kenya. The objective was to determine the accuracy of self-reported MC status in communities where MC for HIV prevention is being rolled out.

**Methods:**

Agreement between self-reported and physically verified MC status was measured among 4,232 men. A structured questionnaire was used to collect data on MC status followed by physical examination to verify the actual MC status whose outcome was recorded as fully circumcised (no foreskin), partially circumcised (foreskin is past corona sulcus but covers less than half of the glans) or uncircumcised (foreskin covers half or more of the glans). The sensitivity and specificity of self-reported MC status were calculated using physically verified MC status as the gold standard.

**Results:**

Out of 4,232 men, 2,197 (51.9%) reported being circumcised, of whom 99.0% were confirmed to be fully circumcised on physical examination. Among 2,035 men who reported being uncircumcised, 93.7% (1,907/2,035) were confirmed uncircumcised on physical examination. Agreement between self-reported and physically verified MC status was almost perfect, kappa (k) = 98.6% (95% CI, 98.1%-99.1%. The sensitivity of self-reporting being circumcised was 99.6% (95% CI, 99.2–99.8) while specificity of self-reporting uncircumcised was 99.0% (95% CI, 98.4–99.4) and did not differ significantly by age group based on chi-square test. Rate of consenting to physical verification of MC status differed by client characteristics; unemployed men were more likely to consent to physical verification (odds ratio [OR] = 1.48, (95% CI, 1.30–1.69) compared to employed men and those with post-secondary education were less likely to consent to physical verification than those with primary education or less (odds ratio [OR] = 0.61, (95% CI, 0.51–0.74).

**Conclusions:**

In this Kenyan context, both sensitivity and specificity of self-reported MC status was high; therefore, MC prevalence estimates based on self-reported MC status should be deemed accurate and applicable for planning. However MC programs should assess accuracy of self-reported MC status periodically for any secular changes that may undermine its usefulness for estimating community MC prevalence in their unique settings.

## Background

Voluntary medical male circumcision (VMMC) program for HIV prevention was launched in Kenya in October 2008 [1, 2] following three randomized controlled trials which demonstrated that male circumcision (MC) reduces the risk of heterosexual HIV acquisition in men by approximately 60% [3,4,5]. Currently, Kenya along with thirteen other sub-Saharan African countries are providing VMMC as a component of comprehensive HIV prevention services and systematically collecting VMMC data as part of their national demographic and health surveys and AIDS indicator surveys [6, 7]. In addition to documenting VMMC program coverage and HIV prevalence, these surveys also monitor MC prevalence across age bands and geographical units [8, 9]. However, the reports from these surveys are usually based on self-reported MC status, which may be prone to misreporting due to people’s tendency to give socially desirable responses. This may manifest as over-reporting of circumcision by uncircumcised respondents in populations where MC practice is the norm and therefore socially desirable or failure of circumcised respondents to disclose their correct status in populations where being circumcised is socially disapproved [10]. Misreporting may also result from men’s uncertainty of their circumcision status due to variation in natural length of the foreskin or because the term “circumcision” may simultaneously refer to foreskin excision and other rituals plus rites of passage in which foreskin may or may not be removed [11].

Using self-reported MC status to estimate MC prevalence, the 2012 Kenya AIDS Indicator Survey reported an increase in MC prevalence in the Nyanza region from 48% in 2007 (before VMMC rollout began) to 66% in 2012, and from 85% to 91% nationally [8, 12]. Among those self-identifying as being a Luo, the largest traditionally non-circumcising ethnic community in Kenya who primarily live in the Nyanza region, MC prevalence was 16.1% in 2007 and increased to 46.7% in 2012 [8]. Substantial rise in MC prevalence estimates based on self-report have also been reported in other African counties including Zambia and Swaziland [13, 14, 15]. Although accurate reporting of circumcision status is critical in monitoring population level MC prevalence and its impact on HIV and other sexually transmitted infections, few studies have validated its accuracy by comparing self-reported and physically verified circumcision status. Results from the few studies that have explored this association are varied [16, 17, 18]. We report on the accuracy of self-reported MC status based on the extent of agreement between self-reported and physically-verified MC status among a large sample of men aged 25–39 years in the Nyanza region of western Kenya.

## Methods

Ethical Approval for this study was obtained from Kenyatta National Hospital/University of Nairobi Ethics Review Committee (KNH-ERC) approval number P36/03/2013 and Centers for Disease Control and Prevention Institutional Review Board (CDC-IRB) approval number 6456.

The agreement between self-reported and physically verified MC status was assessed from May 2014 to June 2015 during a baseline household survey for a cluster randomized trial to determine the effect of interpersonal communication and other interventions to enhance VMMC uptake by men aged 25–39 years in Nyanza region of western Kenya. The survey was conducted in 11 sub counties where 45 locations were selected as study clusters, from which 209 villages were randomly selected for enumeration to identify and mark households where eligible men aged 25–39 years resided.

Male research assistants (RAs) trained on study procedures, including physical verification and documentation of circumcision status, revisited the marked households and administered a written informed consent to eligible men for enrolment in the study and for physical verification of their circumcision status. During consenting, participants were informed that their circumcision status would be verified. The RA then administered a standard questionnaire, which included a question on self-perceived MC status, to each participant in a face-to-face interview immediately after enrolment. At the end of the interview, participants who consented to verification of their circumcision status were asked by the RA to identify a private location where the genital examination could be done. Physically verified circumcision status was recorded as fully circumcised (no foreskin), partially circumcised (foreskin is past coronal sulcus but covers less than one half of the glans) or uncircumcised (foreskin covers one half or more of the glans). The RAs were trained on assessment and classification of MC status by clinicians from a Nyanza-based VMMC implementing partner and another clinician who had participated in an earlier study [16] where circumcision status was physically ascertained within community settings. The clinicians instructed the RAs by first showing them illustrations of the external male genital anatomy and different sizes of foreskins followed by practice on classifying foreskin as fully circumcised, partially circumcised or uncircumcised. RAs used a job aid with photo illustrations of different grades of circumcision during genital examination in order to standardize their classification of MC status.

### Analysis

Descriptive frequencies were generated for demographic characteristics; chi-square tests and proportion tests were used to determine significance when appropriate. Frequency counts and percentages were tabulated for categorical variables. Pearson’s chi-square tests (for categorical variables) were used to determine any statistically significant difference (p<0.05) in demographic characteristics of the reported against the observed circumcision status. To assess the level of agreement between what was reported and what was observed from the physical verification; the kappa coefficient was computed. To verify that the kappa values obtained were not due to chance, p-value was estimated for the kappa estimate. R 3.4.0 statistical package [19] and Stata (*StataCorp*. *2015*. *Stata Statistical Software*: *Release 14*. *College Station*, *TX*: *StataCorp LP)* [20] were used to compute these estimates.

Unadjusted and adjusted logistic regression models were used to assess the demographic predictors of consenting to MC status verification among men aged 25–39 years. We assessed the effect of age, marital status, religion, level of education and employment status of respondents on consenting to physical verification while adjusting for the chance imbalances of all of them. Unadjusted and adjusted odds ratios are reported for ease of interpretation. Trend test was also carried out to detect any monotonic trends within the demographic characteristics.

## Results

Of the 9,679 eligible men enumerated and invited to participate in the survey, 5,639 (58.3%) were enrolled after giving written informed consent; 1,311 declined genital examination. Out of the 4,328 who consented to interview and genital examination, 96 (6.8%) withdrew just before the physical examination and 4,232 (75.1%) agreed to physical verification of their circumcision status and were included in the analysis for agreement between self-reported and physically verified MC status. The characteristics of the participants enrolled in the study are presented in Table 1.

**Table 1:** Characteristics of men enrolled in the study (n = 5,639).

Factor	Consenting to MC Verification		
	Accepted n (%)	Declined n (%)	P-value
**N**	4,232	1,407	
**Age group**			
25–29	1,702 (76.2)	532 (23.8)	0.24
30–34	1,440 (74.0)	507 (26.0)	
35–39	1,090 (74.8)	368 (25.2)	
**Marital status**			
Divorced/Separated/Widowed	113 (83.7)	22 (16.3)	0.04
Married	3,626 (74.6)	1,232 (25.4)	
Single	493 (76.3)	153 (23.7)	
**Religion**			
Christian	4,183 (75.0)	1,391 (25.0)	0.95
Non-Christians	49 (75.4)	16 (24.6)	
**Education completed**			
Primary & below	2,672 (78.7)	725 (21.3)	<0.001
Secondary	1,129 (70.0)	484 (30.0)	
Post-secondary	431 (68.5)	198 (31.5)	
**Employment status**			
Employed	2,537 (72.0)	988 (28.0)	<0.001
Unemployed	1,695 (80.2)	419 (19.8)	
**Self-Reported circumcision status**			
Uncircumcised	2,035 (73.0)	753 (27.0)	<0.001
Circumcised	2,197 (77.1)	654 (22.9)	

Table 2 shows that the 4,232 participants who underwent genital examination for verification of their circumcision status had a substantial match between reported and verified circumcision status for both uncircumcised and circumcised participants, kappa statistic (К) = 98.6% (95% CI, 98.1–99.1). The sensitivity of self-reporting being circumcised was 99.6% (95% CI, 99.2–99.8) while specificity of self-reporting uncircumcised was 99.0% (95% CI, 98.4–99.4) and did not differ significantly by age group based on chi-square test. Similarly, the kappa agreement was almost perfect for all age groups; range 98.1%–99.1%. Of those who reported being circumcised, 99.0% were confirmed as fully circumcised on genital examination (positive predictive value) while 99.6% of those reporting being uncircumcised were confirmed uncircumcised (negative predictive value). On physical examination about 3% (138 out of 4,232) of the men were found to be partially circumcised and 119 (86.2%) of them self-reported that they were uncircumcised. For the purpose of this analysis, partially circumcised men were finally re-classified as uncircumcised in line with the study protocol which recommended that they be treated as uncircumcised and referred for MC.

**Table 2:** Self-reported versus verified circumcision status (n = 4,232).

Factor	Self-Reported Status	
	Circumcised n (%)	Uncircumcised n (%)
**N**	2197	2035
**Findings on Visual inspection of the penis (in a flaccid state)**		
Fully Circumcised	2176 (99.0)	9 (0.4)
Partially Circumcised	19 (0.9)	119 (5.8)
Uncircumcised	2 (0.1)	1907 (93.7)

Men’s readiness to consent for physical verification of MC status differed by client characteristics. Table 3 shows the results of logistic regression from both unadjusted (univariate) and adjusted (multivariate) analysis of demographic variables for association with consenting to genital examination. The proportion of men aged 25–29 years who consented to genital examination was 76.2% compared to 74.8% for men 35–39 years. In the unadjusted analysis, marital status was not a significant predictor, but Divorced/Separated/Widowed men had a higher likelihood of consenting to genital examination (OR = 1.75, 95% CI: 1.10–2.77) compared to married men. Religion (p = 0.950) and age group (p = 0.24) were not significant predictors of consenting for genital examination, however age group was included in the final model as a known confounder. Employment status was associated with consenting to physical verification (p<0.001). In the adjusted analysis, unemployed men were more likely to consent to physical verification (OR = 1.48, 95% CI 1.30–1.69) compared to employed men holding all other predictors constant. Men with secondary and post-secondary education were less likely to consent to genital examination (OR = 0.65, 95% CI: 0.57–0.75) and (OR = 0.61; 95% CI: 0.51–0.74) respectively compared to those with Primary education and below holding all other predictors constant.

**Table 3:** Demographic predictors for consenting to genital examination to verify circumcision status (n = 5,639).

Covariate	Univariate Analysis			Multivariate Analysis		
	OR	95% CI	p-value	OR	95% CI	p-value
**Age group**						
25–29*	1			1		
30–34	0.89	(0.77–1.02)	0.10	0.89	(0.77–1.03)	0.11
35–39	0.93	(0.79–1.08)	0.32	0.92	(0.79–1.08)	0.32
**Marital Status**						
Married *	1			1		
Divorced/Separated/Widowed	1.75	(1.10–2.77)	0.02	1.58	(0.99–2.51)	0.05
Single	1.09	(0.90–1.33)	0.36	1.11	(0.9–1.36)	0.33
**Religion**						
Christian*	1					
Non-Christians	1.02	(0.58–1.80)	0.95			
**Education**						
Primary & below*	1			1		
Secondary	0.63	(0.55–0.72)	<0.001	0.65	(0.57–0.75)	<0.001
Post-Secondary	0.59	(0.49–0.71)	<0.001	0.61	(0.51–0.74)	<0.001
**Employment Status**						
Employed*	1			1		
Unemployed	1.58	(1.38–1.79)	<0.001	1.48	(1.30–1.69)	<0.001

*Reference category

## Discussion

The accuracy of self-reported MC status was notably high in this traditionally non-circumcising community, where agreement between self-reported and physically verified circumcision status was nearly perfect (К = 98.6% (95% CI, 98.1–99.1). The high level of agreement is likely because the study was conducted in a traditionally non-circumcising community where circumcision has been introduced and accepted as a medical intervention devoid of societal norms that may be stigmatizing. These results are comparable to those reported by an earlier study conducted in the same community by Westercamp et al. in which agreement between self- reported and physically verified MC status was 97.4% [18] and one in Zambia and Swaziland where, depending on study site, agreement was 99% with 2–7% of uncircumcised men reporting being circumcised and 0.05–5% of circumcised men reporting being uncircumcised [21].

On the other hand, our findings contrast the results from a study in Lesotho, in which 23.4% of circumcised men misreported their circumcision status [22]. It is possible that over reporting of MC status in Lesotho is related to social desirability bias, given that Lesotho is largely a circumcising country hence men’s tendency to want to be identified as circumcised. The difference between our results from a non-circumcising community and the Lesotho study may also be due to uncertainty over the final circumcision status among men circumcised in the traditional context, because men who participate in traditional circumcision ceremonies that do not involve the removal of the foreskin may presume being circumcised when they are actually not. This has been illustrated by Hewett et al. [21] in Zambia and Swaziland where only 3.3% of men who reported being circumcised by clinicians in medical facilities were found to be uncircumcised on physical verification compared with 41.4% misreporting among men circumcised traditionally. Similarly, 73.3% of the men who reported being circumcised medically in Lesotho were confirmed as circumcised while only 27.6% of those reporting being circumcised traditionally were fully circumcised on examination.

An important factor that may also contribute to misreporting of circumcision status, but did not play a significant role in our study, is partial circumcision. In our study, about 3% (138) of the men were found to be partially circumcised on physical verification and 86% (119) of them self-reported as uncircumcised. The overall direction of reporting partial or incomplete circumcision status among our study participants was towards being uncircumcised. Given that the study was conducted in a traditionally non-circumcising community where medical circumcision had recently been rolled out and most men circumcised as adults, the 119 partially circumcised individuals who self-identified as uncircumcised were likely cases of short foreskin from birth. On the other hand, it is reasonable to presume that 19 partially circumcised men who self-reported being circumcised were cases of incomplete removal of the foreskin during surgery. Intuitively, men with incomplete foreskins after medical circumcision in adulthood would be expected to know and report that they have been circumcised while men with short foreskins from birth would naturally self-report lack of circumcision. It was not possible to compute misreporting of partial circumcision status among our study participants because they only had yes/No options for self-reporting their circumcision status. In the Zambia and Swaziland, study misreporting of partial circumcision status was <2% in urban sites and 5% in rural sites [21]. However, a study in Lesotho [22] reported that 10.0% of the men were found to be partially circumcised, with little difference in misreporting between those who were circumcised medically and those circumcised traditionally.

In addition to measuring the agreement between self-reported and physically verified MC status among men aged 25–39 years, our study provided important insights into factors that influence men’s willingness to undergo physical verification of their circumcision status and MC prevalence among men in the study population. Three-quarters (75.1%) of the men in this study consented to having their circumcision status verified through genital examination by a trained male RA.

There was differential refusal to genital examination by circumcision status (23% among those reporting being circumcised and 27% among those reporting being uncircumcised, P < 0.001) may be linked to embarrassment associated with exposing uncircumcised penis in a community where MC is accepted as a medical intervention. Similarly, employed men were less likely to consent to physical verification than unemployed men. Thus, the rate of accepting physical verification of circumcision status may vary in different communities depending on the distribution of these determinants. Overall consenting rate in our study is comparable to 76% adolescent and 71% adult men who accepted genital examination to verify their MC status in urban Zambia but less than among men in urban Swaziland and rural Zambia where 92–99% accepted verification [21]. A study conducted earlier in our study region by Agot and colleagues in 1999–2000 also demonstrated a high rate of acceptability for physical verification of circumcision status, at 85.1% [19]. Conversely, a study in Kisumu by Westercamp et al. in 2010 at the early stages of VMMC program implementation reported a much lower acceptability of genital examination, at 37% [17].

MC prevalence estimate based on self-report and on physical verification of circumcision status of 4,232 (43.7%) out of 9,679 eligible men enumerated in our study were similar at 51.9% and 51.4%, respectively. These estimates may not be applicable to the parent population because we do not know the profile of 56.3% eligible men who were enumerated but did not participate in the study. The estimates may therefore be unrepresentative if individuals of a given circumcision status were under-represented in the study population due to skewed self-selection of men who did not participate in the survey.

Our study had several limitations. We only provided “Yes” and “No” responses for circumcision status, so we were unable to record responses for participants who may not have known their status or may have reported partial circumcision. This limitation was mitigated by the low proportion (3%) of partially circumcised men who may have been uncertain of their circumcision status and by an overall tendency towards correctly reporting short foreskins from birth as uncircumcised. In less favorable circumstances, such men may arbitrarily assign themselves a status rather than say that they do not know. This was previously observed in a Zambian/Swaziland study [21] in which “don’t know” response was more frequent in ACASI than in face-to-face interview. In another study where “don’t know” was an option, 23% of circumcised and 31% of uncircumcised adolescent men reported not knowing their circumcision status [23].

A further limitation is that participants who were examined knew shortly before the procedure (during the informed consent process) that their circumcision status would be physically verified later, and were likely motivated to report the accurate status. Agreement between self-reported and physically verified circumcision status was possibly overestimated for older and less educated men because a higher proportion of them declined physical verification; Intuitively, men who would have falsely reported being circumcised because of social desirability would be most likely to refuse physical verification. Assuming a worst case scenario in which all men who declined physical verification were uncircumcised ones destined to give a false report of being circumcised, we end up with the worst possible agreement rate of 75% while the best agreement rate remains 99%. But, because circumcision is not a cultural or religious practice in study community, there was no strong motivation for participants to over- or under-report their circumcision status. Studies among traditionally or religiously circumcising communities may over-report being circumcised and yield different results. Finally, if an estimate of MC prevalence from this study is applied to the population it would be based only on 43.7% of eligible men enumerated in the study clusters. The sub-optimal participation rate however did not markedly affect our agreement analysis for self-reported and physically verified circumcision status because participants declined participation overall before being informed of physical verification at enrolment.

Despite these limitations, the study had several strengths pertaining to evaluation of agreement between self-reported and physically verified circumcision status. The loss of men who declined participation before being requested to consent for physical verification of MC status did not introduce any significant bias in the data. Overall, a large proportion (75%) of participants consented to verification of their MC status, thus forming as good basis for our conclusion regarding accuracy of self-reported MC status. Also, due to intense VMMC education as a health intervention in the study region since 2008, it is understandable that most men self-report correctly devoid of cultural norms or pressure. Additionally men in Nyanza are probably well educated about MC both in the context of the randomized trial of MC for HIV prevention conducted in Kisumu in early 2000s and other operational research activities conducted in the region in the initial stages of program roll out. All of these could contribute to explaining why self-report is accurate among these men.

## Conclusions

In conclusion, our study confirmed that in assessing individual circumcision status in the Nyanza region, self-reported circumcision status is accurate, therefore MC prevalence estimates based on self-reports should be deemed appropriate and applicable for planning. VMMC programs should however assess accuracy of self-reported MC status periodically for any secular changes that may undermine its usefulness in estimating community MC prevalence.

## Supporting information

S1 Table Dataset. (CSV)
